# Protein Design with Fluoroprolines: 4,4‐Difluoroproline Does Not Eliminate the Rate‐Limiting Step of Thioredoxin Folding

**DOI:** 10.1002/cbic.202100418

**Published:** 2021-10-08

**Authors:** Jennie O' Loughlin, Silvia Napolitano, Marina Rubini

**Affiliations:** ^1^ School of Chemistry University College Dublin Belfield Dublin 4 Ireland; ^2^ Department of Molecular Biology and Biophysics ETH Zürich Otto-Stern-Weg 5 8093 Zürich Switzerland

**Keywords:** 4,4-difluoroproline, protein design, protein folding, protein stability, thioredoxin fold

## Abstract

C^4^‐substituted fluoroprolines (4*R*)‐fluoroproline ((4*R*)‐Flp) and (4*S*)‐fluoroproline ((4*S*)‐Flp) have been used in protein engineering to enhance the thermodynamic stability of peptides and proteins. The electron‐withdrawing effect of fluorine can bias the pucker of the pyrrolidine ring, influence the conformational preference of the preceding peptide bond, and can accelerate the *cis*/*trans* prolyl peptide bond isomerisation by diminishing its double bond character. The role of 4,4‐difluoroproline (Dfp) in the acceleration of the refolding rate of globular proteins bearing a proline (Pro) residue in the *cis* conformation in the native state remains elusive. Moreover, the impact of Dfp on the thermodynamic stability and bioactivity of globular proteins has been seldom described. In this study, we show that the incorporation of Dfp caused a redox state dependent and position dependent destabilisation of the thioredoxin (Trx) fold, while the catalytic activities of the modified proteins remained unchanged. The Pro to Dfp substitution at the conserved *cis*Pro76 in the thioredoxin variant Trx1P did not elicited acceleration of the rate‐limiting *trans*‐to‐*cis* isomerization of the Ile75‐Pro76 peptide bond. Our results show that pucker preferences in the context of a tertiary structure could play a major role in protein folding, thus overtaking the rules determined for *cis*/*trans* isomerisation barriers determined in model peptides.

## Graphical abstract

**The incorporation** of 4,4‐difluoroproline into the conserved *cis* prolyl‐peptide bond of the *E. coli* thioredoxin variant Trx1P neither eliminated the bottleneck of the thioredoxin refolding reaction nor had an impact on the attainment of the *cis/trans* equilibrium in the unfolded state. Our results suggest that the *cis/trans* isomerisation rates predicted for fluorinated proline analogues in the context of short model peptides are not applicable in the context of tertiary structures.

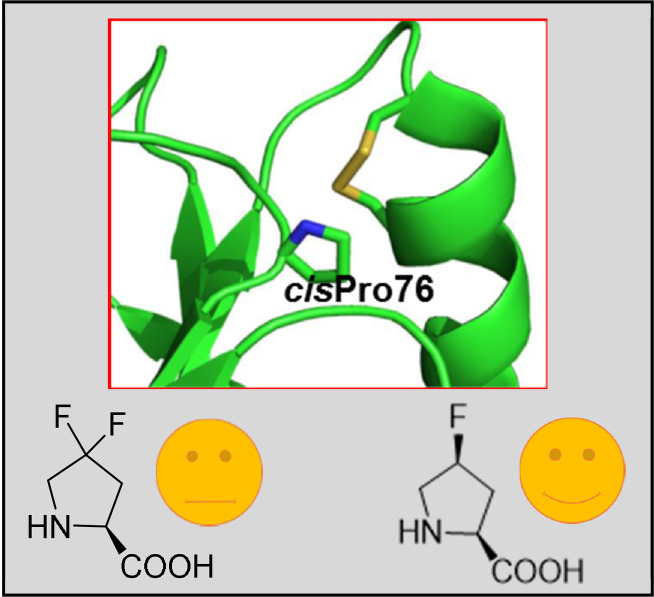

## Introduction

Proline displays unique structural features among the proteinogenic amino acids. Two of the main chain atoms of Pro, C^α^ and nitrogen, are constricted within the pyrrolidine ring, thus conferring conformational restriction on the phi angle in the peptide bond.^[1]^ In folded proteins, although the *trans* conformation of peptide bonds is still slightly favoured between any residue and the following proline (Xaa‐Pro), the steric hindrance of the pyrrolidine ring is not fully alleviated by the adoption of the *trans* conformation. Thus, *cis* and *trans* conformations of Xaa‐Pro peptide bonds are almost isoenergetic.^[2]^ In the cellular environment, the *trans* to *cis* isomerisation of prolyl peptide bonds where Pro is in the *cis* conformation in the native state is a slow process in the time range of several minutes.^[3]^ In nature, peptidyl‐prolyl *cis*‐*trans* isomerases (PPIases) catalyse the formation of the correct peptide conformation by diminishing the energy barrier between the two conformational states.^[4]^ In living organisms, the switching between *cis* and *trans* conformation catalysed by PPIases can act as a molecular timer that regulates the timing of key biological processes.^[5]^ *Trans* to *cis* isomerisation is often the rate limiting folding step in the *in vitro* refolding process for proteins displaying a Pro residue in the *cis* conformation in the native state.^[3]^ The pyrrolidine ring of Pro generally adopts either the *exo* or the *endo* pucker conformation. In the *exo* pucker the C^4^ atom points towards the opposite side of the carbonyl group of Pro, while in the *endo* pucker the C^4^ atom and the Pro carbonyl group are on the same side of the plane. The analysis of a set of high resolution crystal structures of proteins revealed that 89 % of Pro residues involved in a *cis* peptide bond displays the *endo* pucker, while there is no defined preference for one conformation over the other when Pro is found in a *trans* peptide bond.^[6]^

Substitutions on the pyrrolidine ring with electron‐withdrawing or sterically hindered chemical groups can have an influence both on the puckering preference and on the *cis*/*trans* equilibrium.^[7]^ In the context of globular proteins, (2*S*; 4*R*)‐fluoroproline ((4*R*)‐Flp) and (2*S*; 4*S*)‐fluoroproline ((4*S*)‐Flp) have been employed for the fine tuning of protein thermodynamic stability, folding, and bioactivity.^[8]^ Increase in the thermodynamic stability of peptides and proteins substituted with 4‐fluoroprolines have been commonly attributed to the biasing of the ring pucker elicited by the stereochemistry of the electron‐withdrawing fluorine accompanied by favourable pre‐organization effects on the main chain.^[9]^ (4*R*)‐Flp has been shown to promote the *exo* pucker and to stabilise the *trans* peptide bond conformation, while (4*S*)‐Flp favours the *endo* pucker and increases the stability of the *cis*‐peptide bond.^[10]^ In addition, the presence of an electron‐withdrawing element at this position decreases the double bond character of the peptide bond, thus facilitating the *cis*/*trans* isomerization process by diminishing the rotational barrier between the two conformations.^[11]^

The impact of the incorporation of (2*S*)‐4,4‐difluoroproline (Dfp) on the thermodynamic stability and folding kinetics of globular proteins has been by far less investigated. Similar to Pro, Dfp does not seem to have a strong conformational preference, but due to the presence of two electron‐withdrawing fluorine atoms it can enhance the *cis*/*trans* isomerization kinetics to a larger extent than (4*R*)‐ and (4*S*)‐Flp, by diminishing the double bond character of the peptide bond.^[12]^ Therefore, it has been suggested that Dfp might prove useful for enhancing the folding rates of proteins where cis/*trans* isomerisation around the prolyl peptide bond is the rate limiting step. Differently from hydrocarbons in Pro, the fluorocarbon bonds on Dfp display an inverted polarity at the C^4^, thus creating a negatively charged surface. Moreover, Dfp is slightly more lipophilic than Pro.^[13]^ Therefore, Dfp might be also employed to modulate hydrophobic effects in proteins without altering the native conformational preference of the protein.^[14]^

**Figure 1:**
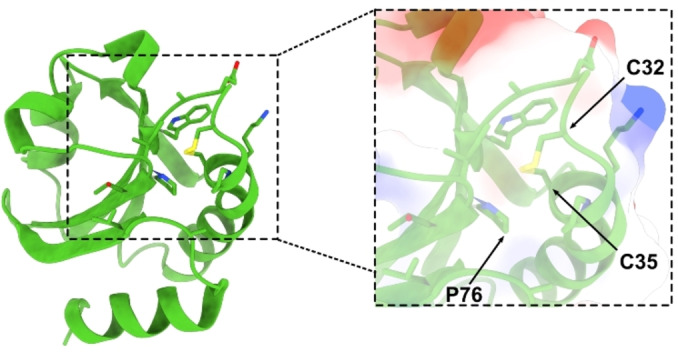
Three‐dimensional structure of *E. coli* thioredoxin (PDB: 2TRX). In the black square is shown the local structure around the catalytic disulfide bond with *cis*Pro76 in close proximity (surface polarity is colour‐coded from negative charge (red) to positive charge (blue)).

In previous studies, we have incorporated (4*R*)‐ and (4*S*)‐Flp into an *E. coli* thioredoxin variant which contains *cis*Pro76 as the only proline residue (Trx1P), whereas the *trans* Pro residues at positions 34, 40, 64, 68 were mutated to Ala.[^8d^, ^8e^] *E. coli* thioredoxin is regarded as the prototype of the family of thioredoxin‐like oxidoreductases. It displays a conserved *cis* prolyl peptide bond at Pro76 (essential for Trx bioactivity and thermodynamic stability) buried in the hydrophobic interior in close proximity to the catalytic disulfide bond between Cys32 and Cys35 (Figure 1).^[15]^ We have shown that the incorporation of (4*S*)‐Flp into Trx1P (Trx‐4*S*‐Flp) accelerated the formation of the native *cis*‐peptide bond in the context of the tertiary structure by nine‐fold relative to the parent protein. In the unfolded state, (4*S*)‐Flp also accelerated the *trans*‐to‐*cis* reaction by one order of magnitude. The incorporation of (4*R)*‐Flp at *cis*‐Pro76 into Trx1P (Trx‐4*R*‐Flp) elicited no changes in the refolding kinetics of the protein, while a two‐fold acceleration of the *trans*‐to‐*cis* reaction was reported in the unfolded state (Table S1 Supporting Information). In this work, we aimed at investigating the effect of Dfp, on the thermodynamic stability and bioactivity of Trx as well as on the kinetics of prolyl *cis*/*trans* isomerization in the context of the intact tertiary structure and in the unfolded state of Trx1P. We reasoned that incorporating a 4‐fluorinated Pro analogue devoid of a strong conformational preference would let us dissect the effects arising from the ring pucker preference and from the electron‐withdrawing effect of fluorine.

## Results and Discussion

The fluorinated protein variant Trx1Dfp, substituted at *cis*Pro76, was produced in M9 minimal medium using the proline auxotrophic cell strain CAG18515 transformed with an additional plasmid for overexpression of an engineered *E. coli* prolyl‐tRNA‐synthetase, ProRS (C443G), to increase Dfp incorporation.^[16]^ After purification by ion exchange chromatography, the yields for the fluorinated protein variant were 5.4 mg/L of bacterial culture. The identity of the isolated protein and the incorporation yields for Dfp (93 %) were confirmed by mass spectrometry (Supporting Information, Figure S1).

**Figure 2:**
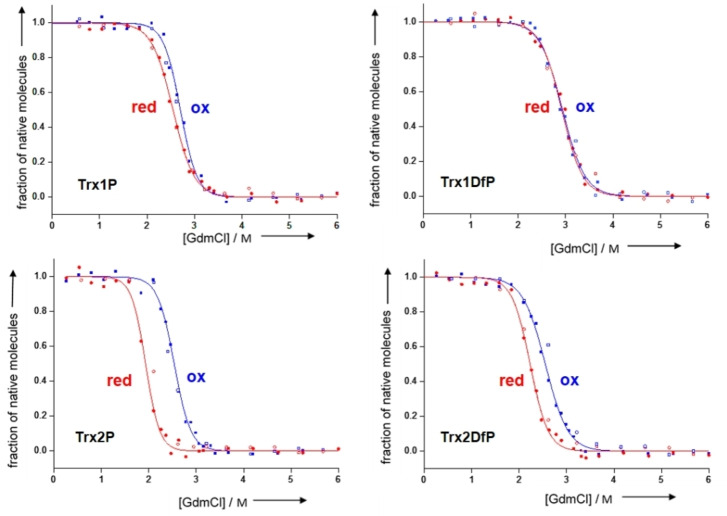
GdmCl‐dependent unfolding‐refolding equilibria of the oxidised (blue) and reduced (red) forms of Trx1P and Trx1Dfp in 50 mM MOPS**⋅**NaOH at pH 7.0 and 25 °C, determined by following the signal change at 220 nm by CD. The unfolding (closed symbols) and refolding (open symbols) equilibria were evaluated according to the two‐state model of folding and normalized (solid lines).

At first, we determined the thermodynamic stability of the oxidised and reduced forms of Trx1Dfp by measuring the unfolding/refolding equilibrium transitions at different guanidinium chloride (GdmCl) concentrations. The free energy of folding (ΔG^0^) for the oxidized and reduced forms of Trx1P were −38.0±1.3 kJ/mol and −29.2±1.7 kJ/mol respectively and were in agreement within experimental error with data previously reported by our group under identical conditions.^[17]^ The thermodynamic stability of the oxidised form of the Trx1Dfp variant (Trx1Dfp_ox_) was significantly decreased (−29.1±1.2 kJ/mol) in comparison to the parent protein, while the ΔG^0^ of the reduced form (Trx1Dfp_red_) was almost identical to that of Trx1P (Figure 2 and Table 1). Therefore, Trx1Dfp_ox_ and Trx1Dfp_red_ displayed roughly the same thermodynamic stability. This is surprising because Trx is an oxidoreductase acting as a strong cytoplasmic reductant and its oxidized form in nature is more stable than the reduced one. The less negative ΔG^0^ of Trx1Dfp_ox_ might arise from the presence of the two electronegative substituents on the C^4^ on the Pro ring, as the pK_a_ value of Cys32 might decrease, thus leading to a destabilisation of the oxidised form of Trx1Dfp at pH 7.0.

**Table 1:** Thermodynamic parameters for the Trx variants determined by unfolding/refolding equilibrium transitions in GdmCl (pH 7.0, 25 °C).

	ΔG^0^_ox_ [kJ/mol]	ΔΔG^0^_ox/red_ [kJ/mol]	m_eq_ [kJ/(mol M)]	Transition midpoint (M GdmCl)
Trx1P_ox_	−38.0+1.3	8.8	14.2+0.9	2.68
Trx1P_red_	−29.2+1.7		11.7+0.4	2.55
Trx1Dfp_ox_	−29.1+.1.2	−0.7	10.1+0.6	2.88
Trx1Dfp_red_	−29.8+1.8		10.3+0.4	2.89
Trx2P_ox_	−36.7+1.7	6.6	14.2+1.1	2.58
Trx2P_red_	−30.1+1.2		15.2+1.2	1.98
Trx2Dfp_ox_	−30.0+1.1	0.9	11.4+1.2	2.63
Trx2Dfp_red_	−29.1+1.3		13.0+1.0	2.24

[a] A negative value of ΔΔG^0^_ox/red_ means that the reduced form of the Trx variant is more stable than the oxidised form.

Next, we determined the redox potential of Trx1Dfp, calculated from the deduced equilibrium constants with wild‐type Trx (E^0^’=‐270 mV) as previously described (Equations 1 and 2 and Figure S2 in the Supporting Information).^[8d]^ The calculated redox potential for Trx1Dfp (E^0^’=‐234 mV) was almost identical to that of the parent protein. Thus, the loss in thermodynamic stability of Trx1Dfp_ox_ did not translate into a more oxidising redox potential.

In order to verify if the destabilising effect of Dfp on the oxidised form of the Trx variant is position dependent, we incorporated a second Dfp at Pro34 into an *E. coli* Trx variant (Trx2P) containing the Pro residues *cis*Pro76 and *trans*Pro34. Pro34 is located between the two catalytic cysteines at the N‐terminal end of α helix 1 and it is solvent exposed, while Pro76 is completely buried inside the tertiary structure (Figure 1).

The yields for the purified protein Trx2Dfp were 3.4 mg/L of bacterial culture and the incorporation yields for Dfp at positions 34 and 76 were ∼89 % (Supporting Information, Figure S3). The free energy of folding for Trx2Dfp, determined from the unfolding/refolding equilibrium transitions, showed that no additional destabilisation occurred upon incorporation of the second Dfp residue (Figure 2 and Table 1). In fact, Trx2Dfp_ox_ was 6.7 kJ/mol less stable than the parent protein Trx2P_ox_, while both proteins displayed a similar thermodynamic stability for the reduced form.

**Figure 3:**
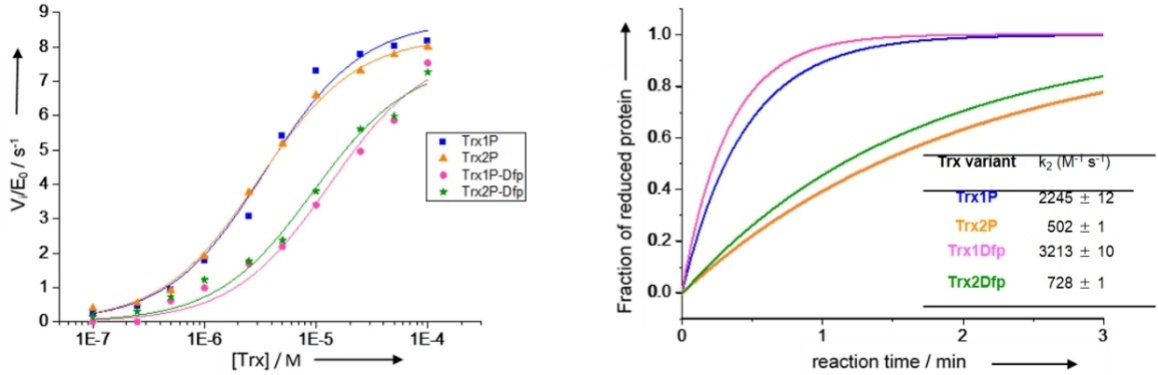
Functional properties of Trx1Dfp and Trx2Dfp compared to their parent proteins Trx1P and Trx2P. Left: Kinetic analyses of Trx variants as substrates of thioredoxin reductase (TrxR) in 50 mM MOPS**⋅**NaOH at pH 7.0 and 25 °C. TrxR activity was determined by monitoring the change in absorbance at 412 nm during the initial 60 seconds of the reaction. Initial velocities were plotted against Trx concentrations and data fitted according to Michaelis‐Menten kinetics (solid lines). Right: Reactivities of the active‐site cysteine pairs in Trx1Dfp and Trx2Dfp compared to Trx1P and Trx2P determined by following the increase in fluorescence at 350 nm (λ_ex_=280 nm) that accompanies the reduction of oxidized Trx variants (2.0 μM) by DTT_red_ (20 nM) at pH 7.0 and 25 °C) (solid lines). Data are an average of three independent measurements.

Next, we investigated the ability of thioredoxin reductase (TrxR) to recognize the Trx variants. In the cellular environment, Trx receives reducing equivalents from NADPH through TrxR, which is the only protein capable of keeping Trx in its active reduced state. *E. coli* TrxR activity was determined by monitoring the increase in absorbance at 412 nm that follows the reduction of 5,5‐dithio‐bis‐(2‐nitrobenzoic acid) (DTNB) by reduced Trx. The initial velocities were plotted against different substrate (Trx) concentrations and evaluated according to Michaelis‐Menten kinetics (Figure 3, left). The K_M_ values for Trx1P and Trx2P were 3.0 μM and 3.5 μM respectively.

The Pro76 to Dfp replacement in Trx1Dfp increased the K_M_ value by 3.1 fold (K_M_=9.5 μM) in comparison to the parent protein, while the additional incorporation of Dfp at Pro34 in Trx2Dfp resulted in a 3.7 fold increase of the K_M_ value (K_M_=13 μM) in respect to the parent protein Trx2P. To determine whether Dfp has an effect on the catalytic activities of the Trx variants, we assessed their ability to act as a reductant of the disulfide bonds of bovine insulin by reduced DTT at different catalyst (Trx) concentrations. The reactions were monitored by following the increase in optical density at 650 nm resulting from aggregation of the reduced insulin B chain (Figure S4 in the Supporting Information).^[18]^ The specific insulin reductase activity of all Trx variants was in the range 4.43 ⋅ 10^−2^–5.68 ⋅ 10^−2^ min^−1^ μM^−1^ and was comparable to that of wild‐type Trx (5.12 ⋅ 10^−2^ min^−1^ μM^−1^).^[18b]^ The fluorinated protein variants were ∼1.2 fold less active than their parent proteins, thus it can be concluded that Dfp has a negligible effect on the catalytic activity of Trx. The reactivity of the disulfide bonds of all variants assessed by the increase in the fluorescence emission at 350 nm (λ_ex_=280 nm) upon reduction of the catalytic disulfides with DTT, showed a 1.5 fold higher reactivity for the fluorinated variants in respect to their parent proteins (Figure 3, right). Therefore, Dfp proved to have only a slightly negative impact on the interaction between Trx and TrxR, but did not affect the catalytic activity or the reactivity of the active‐site disulfide bonds of the fluorinated variants. It is possible that Dfp causes slight changes in the local tertiary structure, in particular at position 76 in the loop facing the active site, thus making it a slightly worse substrate for TrxR.

**Scheme 1:**
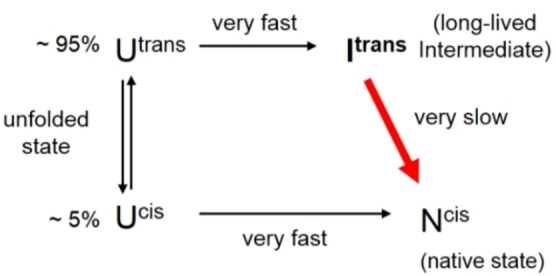
Simplified refolding pathway of Trx1P (adapted from Roderer et al.).^[17]^

Next we investigated the influence of Dfp on the kinetics of proline *cis*/*trans* isomerization in the context of the intact tertiary structure and in the unfolded state of Trx1Dfp. We have previously shown that in the unfolded state, 95 % of Trx1P molecules present a non‐native *trans* peptide bond at Pro76 (U^*trans*^), while 5 % display the native *cis* peptide bond (U^*cis*^).^[17]^ The U^*cis*^ molecules fold very fast to the native state (N^*cis*^), while the 95 % U^*trans*^ collapse into a long‐lived intermediate (I^*trans*^) that displays an intact tertiary structure with a buried non‐native *trans* Pro76. The very slow conversion (t_1/2_=∼100 min) of I^*trans*^ into N^*cis*^ is the bottleneck of the folding pathway of Trx1P (Scheme 1).

We performed interrupted refolding experiments (N‐tests) to determine both the percentage of U^*cis*^ and U^*trans*^ molecules in the unfolded state and the folding kinetics of the I^*trans*^‐to‐N^*cis*^ reaction.^[19]^ Briefly, Trx1P and Trx1Dfp were completely unfolded in GdmCl (4.0 M) at 25 °C and pH 7.0 to reach U^*cis*^/U^*trans*^ equilibrium. The proteins were then let refold at 25 °C by rapid dilution (1 : 20) with MOPS**⋅**NaOH (50 mM, pH 7.0).

**Figure 4:**
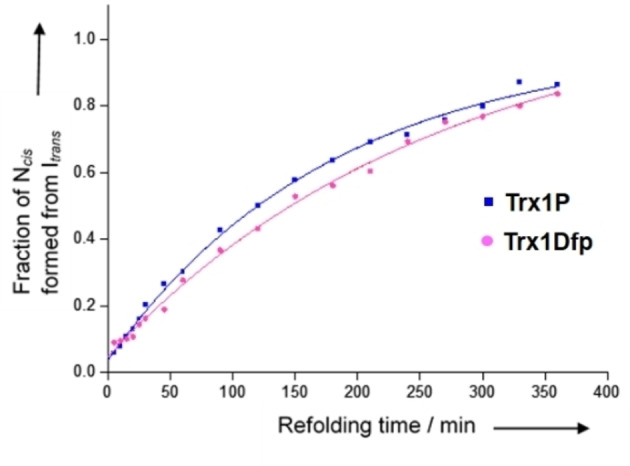
Kinetics of formation of N^*cis*^ from I^*trans*^ during refolding of Trx1P and Trx1Dfp, recorded by interrupted refolding experiments (N‐tests). After complete unfolding in 4.0 M GdmCl, at 25 °C and pH 7.0 the proteins were then let refold at 25 °C by rapid dilution (1 : 20) with MOPS**⋅**NaOH (50 mM, pH 7.0). After different refolding times, the protein samples were unfolded again at 25 °C by addition of GdmCl. This last unfolding step was monitored by following the change in CD signal intensity at 220 nm and the change in the amplitude was plotted against the refolding time. Data are an average of three independent measurements.

Finally, at different refolding times, protein samples were unfolded again at 25 °C by addition of GdmCl (Trx1P: 3.4 M final concentration, Trx1Dfp: 3.2 M final concentration). This final unfolding step was monitored by following the change in CD signal intensity at 220 nm and the change in the amplitude was plotted against the refolding time (Figure 4). The rates obtained from the N‐tests for the I^*trans*^‐to‐N^*cis*^ reaction for Trx1Dfp and the parent protein were almost identical, giving a half‐time of 147±21 min and 124±15 min respectively (Figure 4 and Table 2). The half‐times were also confirmed by fluorescence spectroscopy within the experimental error (Trx1Dfp t_1/2_=143±15 min; Trx1P t_1/2_=128±13 min). In fact, the I^*trans*^‐to‐N^*cis*^ folding reaction can be monitored by following the decrease in the fluorescence emission at 345 nm (λ_ex_=280 nm) when I^*trans*^ is converted to N^*cis*^ (Figure S5 in the Supporting Information). Our results showed that in the context of the tertiary structure of the Trx1P variant, Dfp behaves as (4*R*)‐Flp and Pro and has no effect on the kinetics of the *trans* to *cis* isomerisation reaction.

**Table 2:** Kinetic folding parameters of Trx1P and Trx1Dfp.

	Trx1P	Trx1Dfp
***trans*** **‐to‐*cis* isomerization in the context of tertiary structure**		
k of I_*trans*_→N_*cis*_ [s^−1^]^[a]^	9.28+0.5×10^−5^	7.86+0.7×10^−5^
t_1/2_ of I_*trans*_→N_*cis*_ (min)^[a]^	124+15	147+21

**Ile75‐Pro76** ***cis/trans*** **equilibrium in the unfolded state**		
K_*cis/trans*_^[b]^	0.038+0.009	0.049+0.007
k_*app*_ [s^−1^]^[c]^	2.38+0.48×10^−2^	5.32+0.49×10^−2^
k_*trans to cis*_ [s^−1^]^[d]^	9.04+0.45×10^−4^	2.61+0.21×10^−3^
k_*cis to trans*_ [s^−1^]^[d]^	2.29+0.59×10^−2^	5.06+0.81×10^−2^

**Figure 5:**
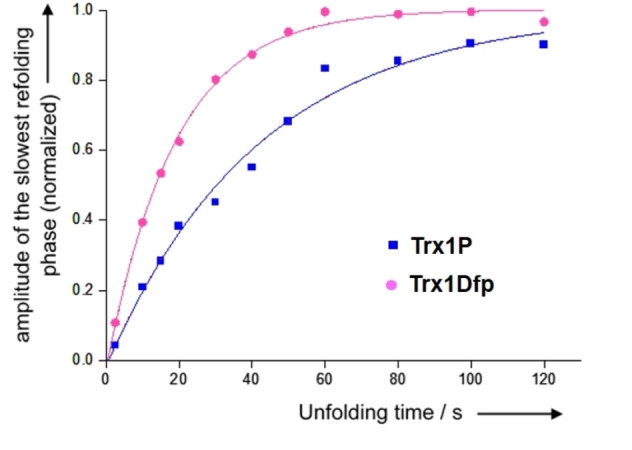
Kinetics of the attainment of the *cis*/*trans* equilibrium of the Ile75‐Pro76 (Trx1P) and Ile75‐Dfp76 (Trx1Dfp) measured by interrupted unfolding experiments (U‐tests). Trx variants were completely unfolded in GdmCl and after different incubation times, proteins were refolded by 50‐fold dilution with MOPS**⋅**NaOH. Refolding was measured by following the decrease in tryptophan fluorescence. The amplitudes of the slow refolding phases (proportional to the fraction of U^trans^ molecules with a *trans* 75–76 peptide bond in the unfolded state prior to refolding) were plotted against unfolding time. Data are an average of three independent measurements.

[a] Values determined by N‐tests (Figure 4). [b] Calculated from the fraction of fast folders (Figure 4). [c] Values determined by U‐tests (Figure 5). [d] Calculated from k_*app*_ and K_*cis/trans*_.

The *trans* to *cis* isomerisation around Tyr47‐Pro48 in the context of a structured intermediate with Pro48 in *trans* is also the rate‐limiting step in the *in vitro* refolding of the barstar protein.^[20]^ Upon incorporation of Dfp into the pseudo wild‐type barstar, containing *cis*Pro48 as single Pro residue, a 3.6 fold increase of the I^*trans*^‐to‐N^*cis*^ folding rate was observed. A similar acceleration (3.5 fold) was reported upon incorporation of (4*S*)‐Flp, while (4*R*)‐Flp at *cis*Pro48 induced no changes in the refolding kinetics relative to the parent protein. As previously mentioned, the I^*trans*^‐to‐N^*cis*^ isomerisation around Ile75‐Pro76 in Trx1P occurs in the context of a fully intact tertiary structure with Pro76 completely buried in the protein interior. In barstar, Pro48 is located at the surface and is largely solvent exposed. These results might suggest that differences in the local polar interactions created by fluorocarbons in the protein core could affect the refolding rates, as these can be influenced by environmental factors.^[21]^ Another plausible explanation might involve the pucker preference of the fluorinated prolines. Bioinformatics analysis has shown that in the thioredoxin fold, there is an almost unique preference for the *endo* pucker of the conserved *cis*Pro in the context of the tertiary structure.^[8d]^ In fact, *cis*Pro76 in Trx1P also adopts the *endo* pucker and a well‐defined *endo* pucker was maintained upon incorporation of (4*S*)‐Flp, while this was less pronounced upon incorporation of (4*R*)‐Flp which favours the *exo* conformation. Thus, we could speculate that no acceleration of the isomerisation rate in Trx1Dfp and Trx‐(4*R*)‐Flp relative to the parent protein is because neither Dfp nor (4*R*)‐Flp favour the adoption of the *endo* pucker. It should be mentioned that when Dfp was incorporated at *cis*Pro32 in human β2‐microglobulin, the *trans* to *cis* prolyl bond isomerisation was no longer the rate limiting step for the folding of this protein.^[22]^ The authors suggested that this behaviour can be attributed to the strong electron‐withdrawing effect of the two fluorine atoms on the Pro ring, as Dfp displays the lowest energy barrier for *cis*/*trans* isomerization in comparison to Pro and its 4‐monofluorinated analogues. Our results show that this explanation does not apply in the context of the thioredoxin fold. It could be speculated that as Pro 32, which displays an *endo* pucker in the crystal structure of β2‐microglobulin, is located in a flexible loop, the biasing of the pucker might play a minor role. Another possible reason that might explain why Dfp did not accelerate the *trans* to *cis* isomerisation rate in Trx1P is the presence of additional energetic costs associated with the protein molecule adopting the *trans*‐amide conformation at position 75–76 in the long‐lived intermediate. It might be speculated that the pucker exerts an influence on the stability of the intermediate itself. In this context, the energetic costs for reaching the native state become identical for Pro and Dfp containing variants.

Next, we investigated the attainment of the *cis*/*trans* equilibrium of the 75–76 peptide bond in the unfolded state of Trx1Dfp by interrupted unfolding experiments (U‐tests).[^19^, ^23^] Briefly, the native protein was unfolded in 5.0 M GdmCl and after different incubation times was then refolded at 25 °C by rapid dilution (1 : 50) with MOPS**⋅**NaOH (50 mM, pH 7.0). The refolding reactions were monitored following the decrease in fluorescence at 345 nm (*λ*_ex_=280 nm) and the refolding amplitudes were plotted against unfolding time and yielded the apparent rate constant (*k*_app_ *=k*_*trans*→*cis*_+*k*_*cis*→*trans*_) of the attainment of the U^*cis*/^U^*trans*^ equilibrium (Figure 5, Table 2). The *trans* to *cis* reaction (*k*_*trans*→*cis*_) for Trx1Dfp was accelerated almost 3‐fold in comparison to Trx1P while the rate constant for the *cis* to *trans* reaction (*k*_*cis*→*trans*_) was ∼2‐fold increased. The increase of both microscopic rate constants *k*_*trans*→*cis*_ and *k*_*cis*→*trans*_ for Trx1Dfp in the unfolded state is slightly lower than the acceleration observed for the model peptides NAc‐Dfp‐OMe and Suc‐Ala‐Ser‐Dfp‐Phe‐pNA in comparison to their correspondent non‐fluorinated peptide (3.5‐5 fold acceleration).^[24]^ The amount of U^*cis*^ molecules for Trx1P determined from the intercepts with the *y*‐axis (N‐tests; Figure 3) was 3.7±0.9 % and was in accordance with our previous studies within the experimental error.[^8e^, ^17^] Trx1Dfp displayed a similar population (4.7±0.7 %) of U^*cis*^ molecules. Therefore, the Pro to Dfp replacement in Trx1P did not significantly change the K_*cis/trans*_ equilibrium (Table 2). The very modest increase in U^*cis*^ molecules in the unfolded state is in agreement with data reported about the population of *cis* conformers in the unfolded state of model peptides where Pro is replaced by Dfp.^[25]^ We have previously shown that (4*S*)‐Flp at position 76 in Trx‐(4*S*)‐Flp accelerated the rate for the *trans* to *cis* isomerisation reaction in the unfolded state by one order of magnitude, while the population of U^*cis*^ molecules was increased to 14 %. Similarly to the results obtained for Trx1Dfp, the incorporation of (4*R*)‐Flp into Trx‐(4*R*)‐Flp led to a 2‐fold increase of the isomerization rate constant *k*_*trans*→*cis*_ and to a slight increase of U^*cis*^ molecules in the unfolded state.

## Conclusion

In conclusion, we have shown that the incorporation of Dfp into the thioredoxin variants Trx1P and Trx2P causes a significant destabilisation of the oxidised form of the fluorinated proteins. The destabilisation was position dependent, suggesting that the substitution at *cis*Pro76 is less well tolerated, likely due to local polarity effects in the hydrophobic protein interior. Further, we proved that the incorporation of Dfp had only minor deleterious effects on the recognition of the fluorinated protein variants Trx1Dfp and Trx2Dfp by TrxR, while the catalytic properties of the variants remained unaffected. Finally, we showed that the replacement of *cis*Pro76 with Dfp did not eliminate the bottleneck of the thioredoxin refolding reaction nor had an impact on the attainment of the *cis/trans* equilibrium in the unfolded state. Our results suggest that the pucker effect in the context of tertiary structures might prevail over *cis*/*trans* isomerisation rates measured for the free proline analogues or in the context of short model peptides.

## Experimental Section

### Equilibrium unfolding and refolding transitions

For the unfolding equilibrium transitions, the various Trx variants were incubated at different GdmCl concentrations overnight at 25 °C, in 50 mM MOPS**⋅**NaOH, pH 7.0. The Trx concentration was 17 μM in all samples. Samples of reduced Trx variants also contained 5 mM DTT. For the refolding equilibrium, the Trx variants were first fully unfolded in 6.0 M GdmCl overnight at 25 °C. MOPS**⋅**NaOH, pH 7.0 was then added, containing different concentrations of GdmCl. These solutions were then incubated at 25 °C overnight, to allow the samples to attain equilibrium. The CD signal of each sample at 220 nm was recorded for 1 min and averaged, on a JASCO J‐810 Circular Dichroism Spectrophotometer. The average CD signals were then plotted against the GdmCl concentrations. The data were fitted according to the two state model of folding and normalized (Equation 1.

(1) $$S{}_{ob}=(m{}_{N}\;\left[D+S{}^{0}{}_{N}\right)+\frac{m_{U}\left[D\right]+S_{U}^{0}-m_{N}\left[D\right]-S_{N}^{0}}{\exp\left(-\frac{\Delta G^{0}+m\left[D\right]}{RT}\right)+1}$$

where m_n_ and m_u_ are the “slopes” of the pre‐ and post‐transition baselines; [D] is the denaturant concentration; *S* ^0^_*n*_ and *S* ^0^_*u*_ are the signals of native and unfolded protein at 0 M denaturant; ΔG^0^ is the free energy of folding at 0 M denaturant; and *m* is the linear dependence of ΔG^0^ on [D]. The values of m_n_ and m_u_ were set to 0 for the fitting.

### Interrupted refolding experiments (N‐tests)

Trx variants were unfolded overnight in GdmCl (4.0 M) at 25 °C and pH 7.0 to attain U^*cis*^/U^*trans*^ equilibrium, and subsequently refolded at 25 °C by rapid dilution (1 : 20) with MOPS**⋅**NaOH (50 mM, pH 7.0) to a final GdmCl concentration of 0.2 M. After different refolding times, samples were unfolded again at 25 °C by addition of GdmCl (Trx1P: 3.4 M final concentration, Trx1Dfp: 3.2 M final concentration). The final Trx concentration was 17 μM. Unfolding was monitored by the change in CD signal intensity at 220 nm on a JASCO J‐810 Circular Dichroism Spectrophotometer. The amplitudes of the unfolding traces, deduced from monoexponential fits with the initial and final signals as open parameters, were plotted against refolding time. Extrapolation of the mono‐exponential fit to zero refolding time yielded the respective fraction of U^*cis*^.

### Interrupted unfolding experiments (U‐tests)

Native Trx variants (N^*cis*^, 200 μM) were rapidly unfolded in GdmCl (5.0 M) with MOPS**⋅**NaOH (50 mM, pH 7.0) at 25 °C, incubated for different times under these conditions, and then refolded at 25 °C by rapid dilution (1 : 50) with MOPS**⋅**NaOH (50 mM, pH 7.0) to a final GdmCl concentration of 0.1 M. The final Trx concentration was 4 μM. The refolding reactions were recorded as the decrease in fluorescence at 345 nm (*λ*_ex_=280 nm), on a Cary Eclipse Fluorescence Spectrophotometer. Refolding kinetics were fitted mono‐exponentially with initial and final signal intensity as open parameters. The refolding amplitudes were plotted against unfolding time and yielded the apparent rate constant (*k*_app_) of the attainment of the U^*cis*^/U^*trans*^ equilibrium.

### Trx variants as a substrate for bacterial TrxR

Experiments were carried out using a quartz cuvette and a Cary‐50 UV‐Visible spectrophotometer, at 25 °C, in 50 mM MOPS**⋅**NaOH, pH 7.0, 0.4 mM EDTA. Recombinant *E. coli* TrxR was incubated with DTNB. Solutions of various concentrations of the Trx variants were added to the TrxR/DTNB solution and the reaction was started by addition of NADPH. The final concentrations were as follows; TrxR 33 nM, Trx 0.1–300 μM, DTNB 3.3 mM and NADPH 0.8 mM. TrxR activity was determined by monitoring the change in absorbance at 412 nm during the initial 60 seconds of the reaction. Slopes were converted into initial velocities using the extinction coefficient ϵ_412nm_=28300 M^−1^ cm^−1^, corresponding to the formation of two TNB molecules per catalytic cycle. Initial velocities were plotted against Trx concentrations and data fitted according to Michaelis‐Menten kinetics.

### Reduction Kinetics of Trx variants by DTT

Equilibrium fluorescence spectra of the Trx variants were recorded between 320 and 400 nm, with excitation at 280 nm, using a Cary Eclipse Fluorescence Spectrophotometer. The reduction of the oxidized form of the Trx variants was measured by addition of reduced DTT with a final concentration of 2 μM Trx and 20 nM DTT in 100 mM NaH_2_PO_4_‐NaOH, pH 7.0. The sample was excited at 280 nm and the increase in emission was recorded at 350 nm. The monoexponential fitting was used to calculate the rate of reduction.

## Conflict of interest

The authors declare no conflict of interest.

## Supporting information

As a service to our authors and readers, this journal provides supporting information supplied by the authors. Such materials are peer reviewed and may be re‐organized for online delivery, but are not copy‐edited or typeset. Technical support issues arising from supporting information (other than missing files) should be addressed to the authors.

Supporting Information
