# Supplementary material for: Protein Design with Fluoroprolines: 4,4‐Difluoroproline Does Not Eliminate the Rate‐Limiting Step of Thioredoxin Folding
Source: Chembiochem. 2021 Oct 8;22(23):3326–32. doi: 10.1002/cbic.202100418 (PMC9292674; doi:10.1002/cbic.202100418)
Supplement: Supplementary file 1 — Supporting Information [file CBIC-22-3326-s001.pdf]

ChemBioChem

Supporting Information

Protein Design with Fluoroprolines: 4,4-Difluoroproline Does Not Eliminate the Rate-Limiting Step of Thioredoxin Folding

Jennie O' Loughlin, Silvia Napolitano, and Marina Rubini*

1. Material and methods

1.1 Materials

1.2 Methods:

1.2.1 Proteins Expression and Purification

1.2.2 Determination of the redox potential of Trx1Dfp

1.2.3 Insulin Reduction Assay

1.2.4. Refolding Kinetics of Trx variants

2. Supplementary Figures and Tables

1 Materials and Methods

1.1 Materials

The following were obtained from Sigma: NADPH tetrasodium salt, 5,5'-dithiobis-2-nitrobenzoic acid (DTNB), insulin from bovine pancreas. (*S*)-4,4-Difluoropyrrolidine-2-carboxylic acid (95% purity) was purchased from Fluorochem. *E. coli* TrxR was a gift from Prof. Rudi Glockshuber (ETH Zurich).

1.2 Methods

1.2.1 Proteins Expression and Purification

Trx1P and Trx2P were produced and purified as described previously.[1]

Trx1Dfp and Trx2Dfp were produced using the proline auxotrophic cell strain CAG18515, co-transformed with the plasmids pGDR11-Trx1P or pGDR11-Trx2P and pTARA-ProRS(C443G). A single colony was inoculated into fresh LB (50 mL) supplemented with the appropriate antibiotics (100 μg/mL ampicillin, 35 μg/mL chloramphenicol) and incubated overnight at 37°C. The overnight cell culture was centrifuged at 4500 rpm for 15 min. The cell pellet was collected and resuspended in 1 L M9 MM (0.3 mM Pro, ampicillin 100 μg/mL and chloramphenicol 34 μg/mL). L-Arabinose (0.1 % w/v) was also added to the medium, to induce the expression of ProRS (C443G). The culture was incubated at 37°C, until cell growth reached a plateau. After centrifugation at 4000 rpm for 15 min, the cell pellet was washed twice with 50 mL 0.9 % NaCl. The cell pellet was then resuspended in 1 L M9 MM (0.6 M NaCl, ampicillin 100 μg/mL and chloramphenicol 34 μg/mL, no proline). The cell culture was incubated for 20 min at 37°C before adding 4,4-difluoroproline (3 mM final concentration). Cell culture was shaken for further 20 minutes at 37°C. IPTG (1 mM) was added and protein production was allowed to proceed for 2.5 hrs at 37°C. Protein purification was carried out exactly as described previously.[1]

The gene sequence for *E. coli* ProRS (C443G) was amplified from plasmid pWK2 and cloned into the pTARA-T7Polymerase plasmid using the restriction sites SacI/XbaI.[2]

1.2.2 Determination of the redox potential of Trx1Dfp

Redox potentials were determined via their equilibrium constant, using Trx wt as a reference, following published procedures.[3] First, Trx wt was freshly reduced in 1 mM DTT and subsequently all Trx variants were buffer exchanged against 50 mM MOPS·NaOH pH 7.0, 1 mM EDTA using PD MiniTrap G-25 columns (GE Healthcare) (500 μL protein load, 200 μL wash and 700 μL elution volume). Oxidized Trx variants were mixed with reduced Trx wt in a final volume of 100 μL (Trx variant 10 μM, Trx wt 10-30 μM), and incubated for 1 day at 25°C to attain equilibrium. The disulfide exchange reaction was quenched by addition of 12 μL formic acid and 50 μL were loaded on a ZORBAX C8 reverse phase column (300 Å, 4.6 x 250 mm,

from Agilent), equilibrated with 35% acetonitrile in water, 0.1% TFA. Protein were eluted with a gradient from 35 to 65% acetonitrile in water, 0.1% TFA over 30 min, with a flow rate of 1 mL/min and a temperature of 70°C. Elution profiles were recorded following the absorbance signal at 220 nm, and protein concentrations calculated via their peak area (using the Peak Analyzer tool of OriginPro 2018b, OriginLab). Redox equilibrium constants were calculated according to Equation 1 and obtained K_{eq} were used to determine the redox potential (E'_0) of each Trx variant, according to Nernst equation (Equation 2), using E'_0 of the Trx wt_{red}/Trx wt_{ox} (-270 mV) redox couple as a reference.[4]

Equation 1: $K_{eq} = \frac{[Trx\ variant_{red}][Trx\ wt_{ox}]}{[Trx\ variant_{ox}][Trx\ wt_{red}]}$

Equation 2: $E'_0 = -270\ mV + (\frac{RT}{2F} lnK_{eq})$

1.2.3 Insulin Reduction Assay

The catalytic activity of the Trx variants on the reduction of bovine insulin by DTT_{red} was measured in 100 mM KH_2PO_4, pH 7.0, 2 mM EDTA. The Trx variants were incubated with DTT_{red} for 5 mins and the reaction was started by addition of the bovine insulin. The final concentrations were as follows: DTT_{red}: 1.67 mM; bovine insulin: 130 µM; and Trx: 0.5, 0.75, 1, 1.25, 1.5, 1.75, 2 µM. The reaction was monitored via the increase in optical density at 650 nm. Aggregation onset was measured as the time taken for the optical density at 650 nm to reach 0.1, using a Jenway 7315 Spectrophotometer. The reciprocal of the aggregation onset was plotted against Trx concentration to obtain linear plots, where the slope of the linear fit was the rate of reaction.

1.2.4. Refolding Kinetics of Trx variants

Trx variants were unfolded overnight in GdmCl (4.0 M) at 25 °C to attain U_{cis}/U_{trans} equilibrium, and subsequently refolded at 25 °C by rapid dilution (1:20) with MOPS·NaOH (50 mM, pH 7.0) to a final GdmCl concentration of 0.2 M. The refolding reaction was followed on a Cary Eclipse Fluorescence Spectrophotometer at 345 nm emission (λ_{ex} = 280 nm). The decrease in emission was plotted against reaction time (min) and fitted monoexponentially to give the rate of I^{trans} to N^{cis} conversion.

2. Supplementary Figures

Supplementary figure 1

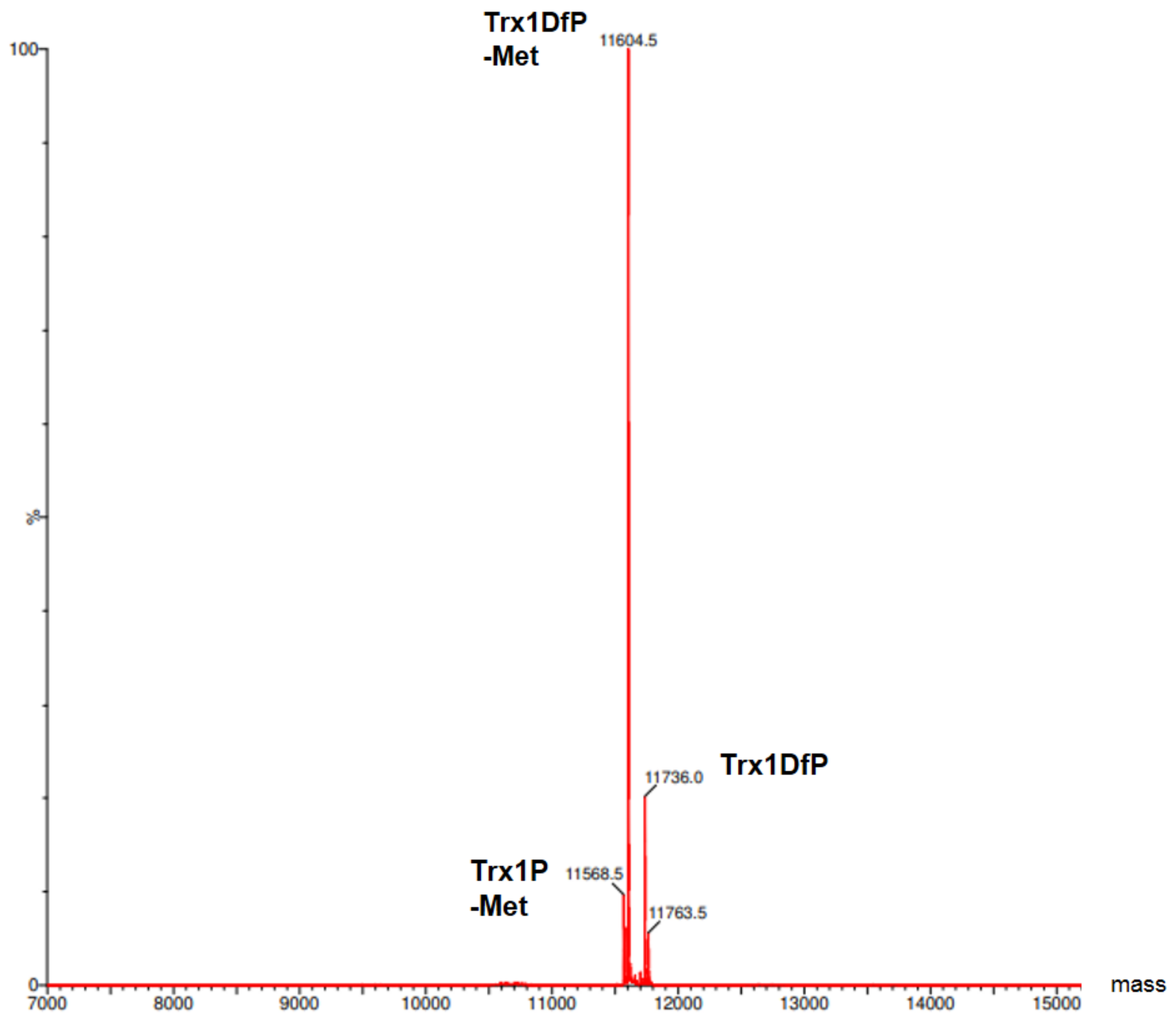

Supplementary Figure 1. Deconvoluted ESI-MS spectrum of *E. coli* Thioredoxin variant Trx1Dfp.

Supplementary figure 2

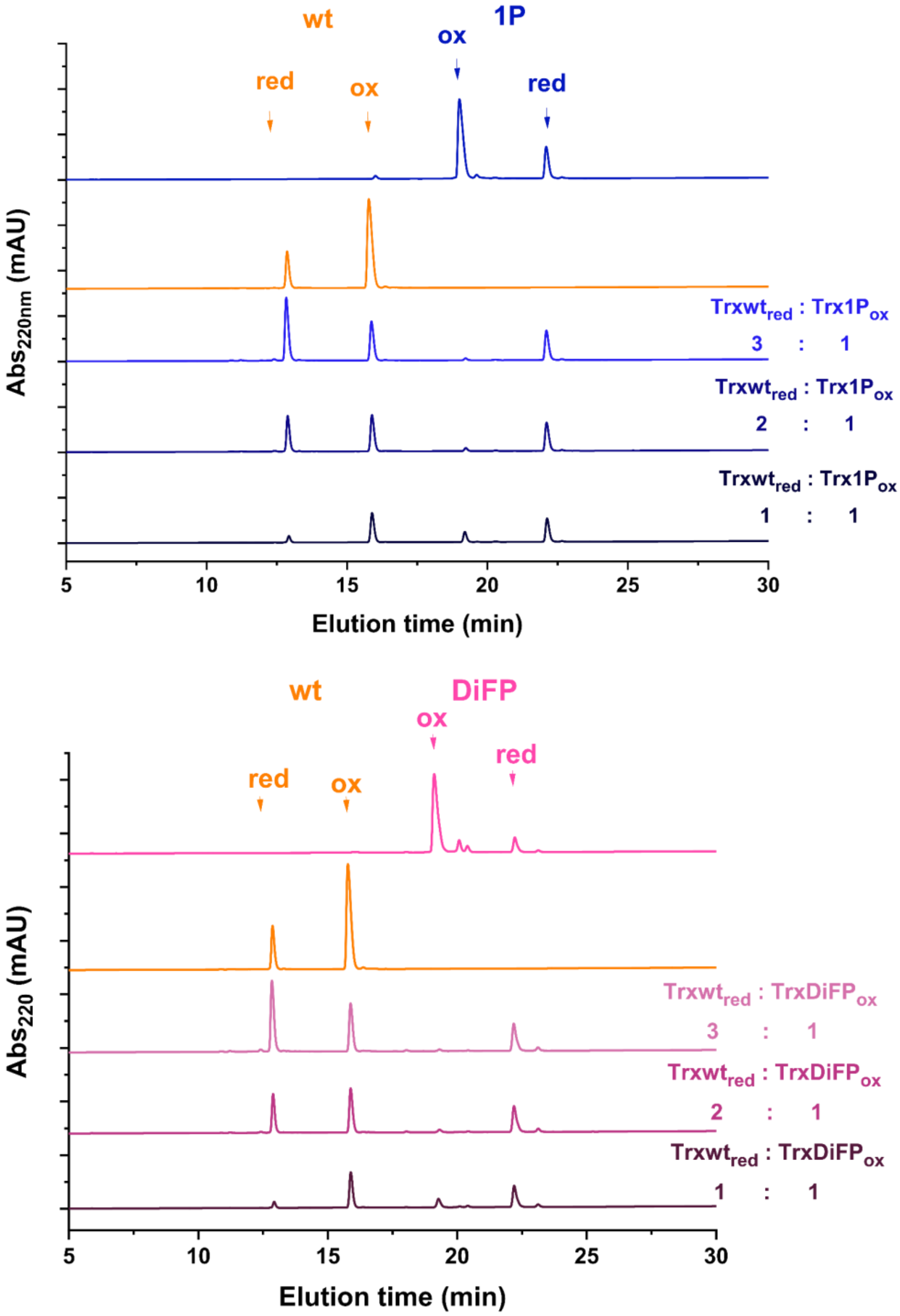

Supplementary Figure 2. Redox equilibria between Trx wt and 1P or DiFP at pH 7.0 and 25°C. Reduced Trx wt was mixed with the oxidized form of the respective Trx variant at a 1:1, 2:1 or 3:1 molar ratios. The reactions were quenched with formic acid, and all redox forms separated by reversed phase HPLC. Peak areas were converted to concentrations, from which redox equilibrium constants (K_{eq}) were calculated (see Equation 1, Materials and Methods section). K_{eq} values were found to be independent of the mixing ratio between reduced Trx wt and oxidized Trx1P or TrxDiFP, showing that the redox equilibria were attained.

Supplementary figure 3

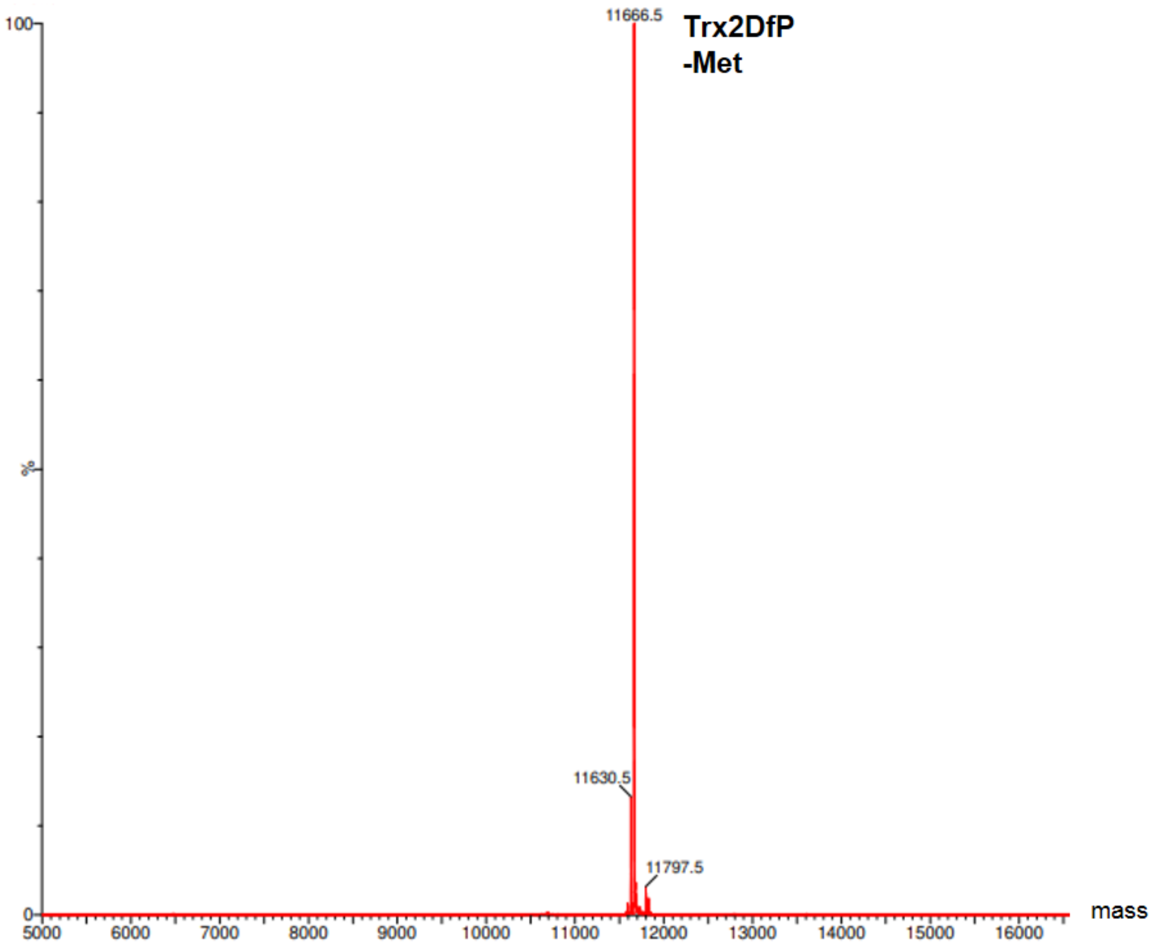

Supplementary Figure 3. Deconvoluted ESI-MS spectrum of *E. coli* Thioredoxin variant Trx2Dfp.

Supplementary figure 4

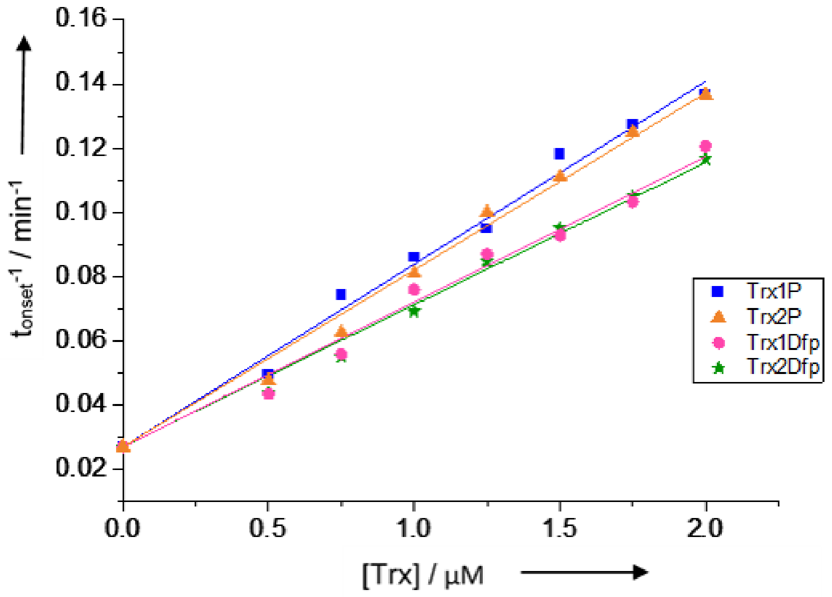

Supplementary Figure 4. Reactivity of Trx variants as reductants of non-natural disulfide substrates at pH 7.0 and 25 °C, specifically as catalysts of insulin (0.13 mM) reduction by DTT (1.67 mM). Reactions were followed by the increase in optical density at 650 nm, caused by aggregation of the reduced insulin B chain. The inverse time of aggregation onset (time required for an increase in OD_{650} of 0.1) depended linearly on catalyst concentration between 0.5 and 2.0 µM Trx.

Supplementary figure 5

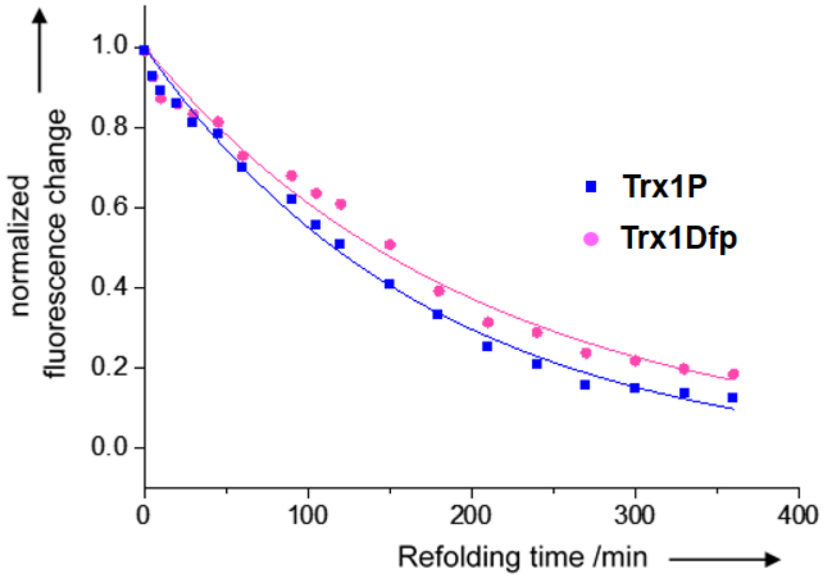

Supplementary Figure 5. Kinetics of the rate-limiting I^{trans}-to-N^{cis} reaction of Trx1P and Trx1Dfp, determined by the decrease in tryptophan fluorescence at 345 nm upon excitation at 280 nm. The rate-limiting phase was fitted monoexponentially. The half-lives were determined to be 128 min for Trx1P and 143 min for Trx1Dfp.

Table S1. Kinetic folding parameters of Trx1P, Trx-(4*R*)-Flp, Trx-(4*S*)-Flp, and Trx1Dfp

	**k of I_{trans}→N_{cis} [s^{-1}][a]** (in the context of tertiary structure)	**$k_{trans\ to\ cis}$ [s^{-1}][b]** (unfolded state)	**Normalised rate for k of I_{trans}→N_{cis}**	**Normalised rate for $k_{trans\ to\ cis}$**
**Trx1P**	$9.3 \pm 0.5 \times 10^{-5}$	$9.0 \pm 0.5 \times 10^{-4}$	1	1
**Trx-(4*R*)-Flp[5]**	$8.7 \pm 2.5 \times 10^{-5}$	$2.0 \pm 0.7 \times 10^{-3}$	0.94	2.2
**Trx-(4*S*)-Flp[5]**	$8.0 \pm 2.5 \times 10^{-4}$	$9.4 \pm 1.0 \times 10^{-3}$	8.6	10.4
**Trx1Dfp**	$7.9 \pm 0.7 \times 10^{-5}$	$2.6 \pm 0.2 \times 10^{-3}$	0.85	2.9

[a] Values determined by N-tests. [b] Calculated from the fraction of fast folders.

References

[1] M. Rubini, M. A. Scharer, G. Capitani, R. Glockshuber, *Chembiochem* **2013**, *14*, 1053-1057.

[2] W. Kim, A. George, M. Evans, V. P. Conticello, *Chembiochem* **2004**, *5*, 928-936.

[3] S. Napolitano, R. J. Reber, M. Rubini, R. Glockshuber, *Journal of Biological Chemistry* **2019**, *294*, 14105-14118.

[4] a) E. C. Moore, P. Reichard, *Journal of Biological Chemistry* **1964**, *239*, 3453-&; b) A. P. Carvalho, P. A. Fernandes, M. J. Ramos, *Prog Biophys Mol Bio* **2006**, *91*, 229-248.

[5] D. Roderer, R. Glockshuber, M. Rubini, *Chembiochem* **2015**, *16*, 2162-2166.
